# Change in Rotavirus Vaccine Coverage in Brazil from before (2015–2019) through the COVID-19 Pandemic Period (2020–2021)

**DOI:** 10.3390/v15020292

**Published:** 2023-01-19

**Authors:** Larissa Lima Barros, Luana Lima Barros, Rodrigo Feliciano do Carmo, Márcio Bezerra Santos, Anderson da Costa Armstrong, Rebeca Araújo de Vasconcelos, Carlos Dornels Freire de Souza

**Affiliations:** 1 Collegiate of Medicine, Federal University of the São Francisco Valley (UNIVASF), Petrolina 56304-917, Brazil; 2 School of Medicine, Faculty of Medicine of Juazeiro do Norte—FMJ/IDOMED, Juazeiro do Norte 63048-080, Brazil; 3 Collegiate of Pharmacy, Federal University of the São Francisco Valley (UNIVASF), Petrolina 56304-917, Brazil; 4 Collegiate of Nursing, Federal University of Alagoas, Arapiraca 57309-000, Brazil

**Keywords:** rotavirus, vaccine, COVID-19, Brazil

## Abstract

During the COVID-19 pandemic, a reduction in vaccination coverage of children and adolescents was observed in several countries. The aim of this study was to assess the impact of the pandemic, in the first two years, on human rotavirus vaccine (HRV) coverage in Brazil compared with previous years. The number of doses of HRV administered in the period from January 2015 to December 2021 and its annual vaccination coverage were analyzed. The vaccination coverage decreased to 77.3% in 2020 and to 70.4% in 2021, substantially lower than the minimum that would be expected (89.2%); the decline was more pronounced in the second year of the pandemic despite the fact that in this period, the circulation restrictions were already less tight. Of the five Brazilian macro-regions, the northeast had the largest decline, and the south had the smallest impact on coverage. At the municipal level, less than half of the Brazilian municipalities managed to achieve vaccination coverage above 90% in either pandemic year. Although there was already a downward trend in coverage in the pre-pandemic years, the present study shows that the values recorded in 2020 and 2021 were significantly lower. Monitoring of vaccination coverage in the coming years should be carried out continuously in order to avoid a possible resurgence of rotavirus-induced diarrhea.

## 1. Introduction

Diarrhea is the fourth leading cause of mortality in children under five years of age worldwide, accounting for approximately half a million deaths in 2019. The highest diarrheal mortality rates are observed in underdeveloped countries in western and central Africa, eastern and southern Africa, and South Asia [1]. In Brazil, in the same year, 3856 deaths from diarrhea and gastroenteritis of presumed infectious origin were recorded, of which about 10% were children under one year of age [2].

The main etiology of diarrheal diseases, for all ages, is rotavirus [3]. Rotaviruses are members of the family Reoviridae and their genome consists of 11 double-stranded RNA segments, which encode six structural and six nonstructural proteins [4]. The virulence of the virus is multigenic and has been associated with genes 3, 4, 5, 9, and 10 [5]. Seven different species of rotaviruses (named A to G) have been linked to infections in various animal species, but only three (A, B, and C) are responsible for infection in humans [6]. Limited infectivity studies indicate that 10 or fewer viral particles are sufficient to result in effective replication [7].

In 2009, the World Health Organization (WHO) recommended that rotavirus vaccines should be included in all national immunization programs and should be considered a priority, particularly in countries with high mortality rates associated with rotavirus gastroenteritis [8]. The introduction of the oral vaccine against human rotavirus (HRV) in Brazil occurred in 2006 through the free provision of two doses, administered at two and four months of age, as recommended by the National Immunization Program (PNI, acronym in Portuguese) [9]. By January 2022, 114 countries have included the rotavirus vaccine in their vaccination routines [10], leading to a substantial reduction in morbidity and mortality due to diarrhea secondary to this pathogen [11].

In Brazil, the PNI was formulated in 1973 and regulated in 1975 by Federal Law No. 6259, with the objective of guiding and systematizing all vaccination actions in the national territory. Throughout its trajectory, the PNI has become an international reference for the control of vaccine-preventable diseases [9]. Approximately 48 immunobiological products are available, 300 million doses are distributed annually, and more than 100,000 professionals work in vaccine rooms [9]. The available rotavirus vaccine is the monovalent (G1P [8]) of the RIX4414 strain (attenuated) [9].

In December 2019, China reported cases of a pneumonia of unknown etiology to the WHO. In January, a new coronavirus was identified as the causative agent [12]. Called SARS-CoV-2 (severe acute respiratory syndrome coronavirus 2) and the resulting disease called COVID-19 (Coronavirus disease 2019), it quickly spread across countries, resulting in a global pandemic with devastating impact on the economy and health services worldwide [12].

During the 2020 pandemic of COVID-19, a reduction in vaccination coverage concerning children and adolescents was observed [13]. The health measures adopted because of the pandemic, such as lockdown, quarantines, and social distancing, despite the recommendation that immunization services should not be interrupted, affected the vaccination actions that required the presence of individuals to the health services [14,15]. In Brazil, from 2019 to 2020, there was a drop in the average vaccination coverage of children up to one year of age of 11.10%, a value that had never been recorded, falling from 84.44% to 75.07% [16]. It is important to highlight that the phenomenon of vaccine hesitancy was already underway in the country, which was negatively impacting vaccination [17]. The spread of fake news in digital media regarding possible side effects and vaccine safety, as well as the pandemic in general, was a potentiator of this already chronic phenomenon [18,19].

Therefore, this paper aimed to analyze the impact of the COVID-19 pandemic on human rotavirus vaccination coverage in the first two years of the COVID-19 pandemic in Brazil.

## 2. Materials and Methods

### 2.1. Study Design, Population, and Period

This is an ecological study involving the number of doses of HRV administered in the period from January 2015 to December 2021 and its annual vaccination coverage. The period from 2015 to 2019 (prior to the COVID-19 pandemic) was used to calculate the expected coverage for the years of 2020 and 2021 (the pandemic period).

### 2.2. Study Area

**Figure 1:**
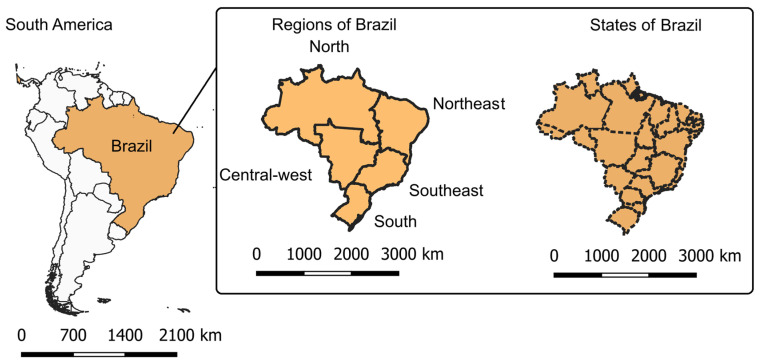
Study area: Brazil and its regions.

Brazil has a territorial area of about 8.5 million km^2^. The country is composed of 26 states and the Federal District, which are divided into five large regions: north, northeast, south, southeast, and center-west (Figure 1). In 2021, the Brazilian population was estimated at 213 million inhabitants, about 14.7 million of which being children under 5 years of age [20]. The country is characterized by important socioeconomic differences: the Municipal Human Development Index (MHDI) for the macro-regions, in 2010, showed that the highest value found was in the southeast, with a value of 0.766; and the lowest was found in the northeast, with a value of 0.663 [21].

### 2.3. Vaccination Coverage

The National Vaccination Calendar for children states that HRV should be administered in 2 doses, with the recommended age for the first dose being 2 months and for the second dose, 4 months. However, the minimum age for the first dose is 1 month and 15 days and the maximum age is 3 months and 15 days, while the minimum age for the second dose is 3 months and 15 days and the maximum age is 7 months and 29 days. The recommended interval between doses is 60 days, 30 days being the minimum [22].

The vaccine is especially effective against serotype G1, although studies indicate cross-protection against other serotypes (G2, G3, G4, and G9). Additionally, it is noteworthy that all these strains are circulating in the world [9,10,11].

Since HRV is administered in more than one dose, the vaccination coverage value is relative to the rate of application of the last dose, thus accounting for total effective immunization [23]. To calculate vaccination coverage, the following equation was considered:

$$\mathrm{Vaccination}\;\mathrm{coverage}\;\left(\%\right)=\frac{number\;doses\;administred\;\left(2nd\;dose\right)}{\mathrm{Target}\;\mathrm{population}\;(<1\;\mathrm{years}\;\mathrm{old})}\times 100$$

The vaccination coverage values are calculated by the Immunization Program Evaluation System, with the number of doses applied coming from the Bulletins of Applied Vaccine Doses and the target population of those under 1 year old taken from the available data on live births obtained from the Information System on Live Births (SINASC, acronym in Portuguese) [23]. For HRV, the Ministry of Health has set a vaccination coverage goal of 90% [24]. Values greater than 100% in vaccination coverage may represent inaccuracies in population estimates and/or in the information regarding the doses administered.

### 2.4. Data Source

The data were collected from the Immunization Program Evaluation System, which records, by age group, the immunobiological doses applied, and also calculates the vaccination coverage by municipality, state, and country. The National Immunization Program (PNI, acronym in Portuguese) was established in 1973 with the main objective of providing all vaccines with quality to all children who are born annually in Brazil. Today, the PNI is an integral part of the World Health Organization’s program, with technical, operational, and financial support from the United Nations International Children’s Emergency Fund (UNICEF) and contributions from the United Nations Development Program (UNDP) [25].

### 2.5. Assessing the Impact of the COVID-19 Pandemic on Vaccine Coverage

To quantify the impact of the COVID-19 pandemic on vaccination coverage, the percentage change was calculated using the following equations:

For 2020:

$$Percentage\;change=\frac{Vaccination\;coverage\;\left(2020\right)-expected\;vaccination\;coverage\;\;\left(mean\;2015\;to\;2019\right)}{expected\;vaccination\;coverage\;\left(mean\;2015\;to\;2019\right)}\times 100$$

For 2021:

$$\begin{array}{c} Percentage\;\;change\\ \;\;\;\;\;\;\;\;\;\;\;\;\;\;\;\;\;\;\;\;\;\;\;\;\;\;\;=\frac{Vaccination\;coverage\;\left(2021\right)-expected\;vaccination\;coverage\;\;\left(mean\;2015\;to\;2019\right)}{expected\;vaccination\;coverage\;\left(mean\;2015\;to\;2019\right)}\\ \;\;\;\;\;\;\;\;\;\;\;\;\;\;\;\;\;\;\;\;\;\;\;\;\;\;\;\times 100 \end{array}$$

where 1—the event analyzed is the vaccination coverage and 2—the expected value for the year is calculated, this being the average of the last five years prior to the start of the pandemic, as recommended [26]. The impact of the pandemic on vaccination coverage is presented in absolute and relative frequencies. Additionally, thematic maps were prepared for the presentation of the results.

### 2.6. Ethical Aspects

This study did not require ethics committee approval since it used secondary data from the public domain, in which there is no individual identification of patients.

## 3. Results

**Figure 2:**
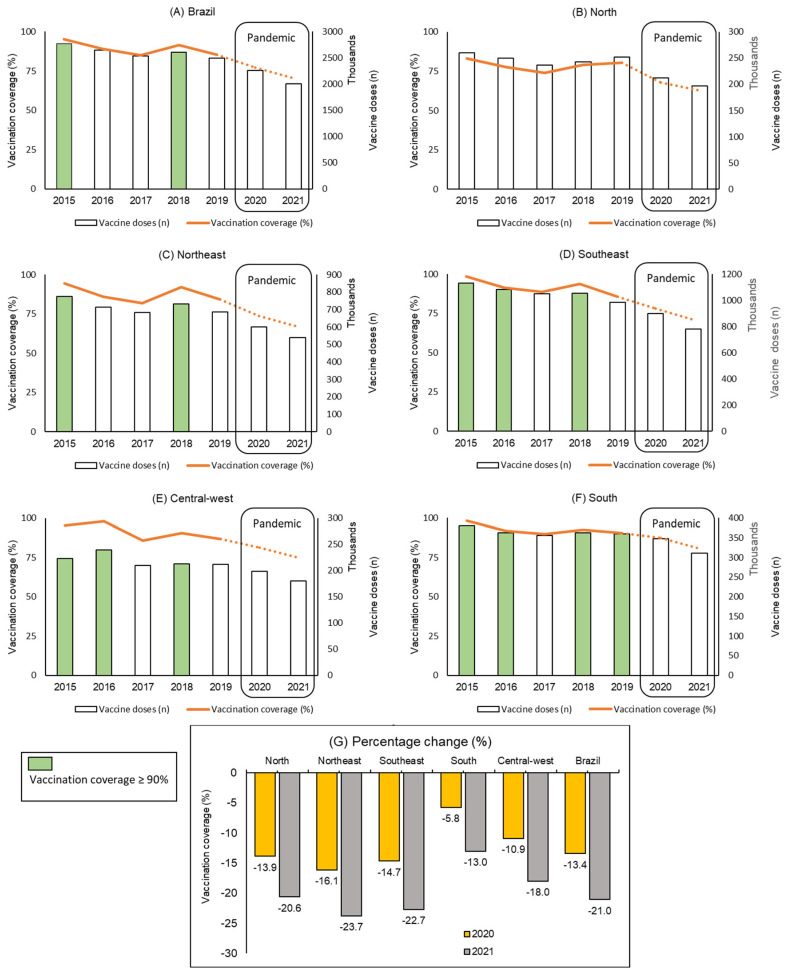
Temporal evolution of human rotavirus vaccination coverage before and during the COVID-19 pandemic in Brazil, according to regions. Brazil, 2015–2021. Figures (**A**–**F**) present the number of second doses applied (columns) in each region of the country and the proportional vaccination coverage achieved (row). The columns in green represent the years in which the regions reached the coverage target recommended by the Ministry of Health (≥90%). Figure (**G**) presents the impact of the COVID pandemic on vaccination coverage in the years 2020 and 2021.

Over the time series, human rotavirus vaccination coverage was more than 90% in the years 2015 (95.35%) and 2018 (91.33%). In the other pre-pandemic years, coverage ranged from 85.1% (2017) to 89.0% (2016). In the pandemic years, the vaccination coverage decreased to 77.3% in 2020 and to 70.4% in 2021, substantially lower than the minimum that would be expected (89.2%). In 2020, a 13.4% reduction was observed, and in 2021, the reduction reached 21.0% (Figure 2).

The northern and northeastern regions presented lower vaccination coverage. The north stands out for never having reached the minimum vaccination goal of 90%. In the northeast, the goal was reached in two pre-pandemic years (2015 and 2018). On the other hand, the southern region of the country was the one with the highest coverage, having reached the goal in four (of the five) pre-pandemic years (Figure 2).

As occurred at the national level, the impact of the pandemic in all regions was greater in the year 2021. In three regions (north, northeast, and southeast), the reduction in vaccination coverage was greater than that observed at the national level. Of these three regions, the northeast experienced the greatest decline (a 16.1% reduction in 2020 and a 23.7% reduction in 2021). In this region, the expected vaccination coverage was at least 87.6%; however, the vaccination coverage observed was 73.5% in 2020 and 66.8% in 2021 (Figure 2).

On the other hand, the southern region had the least impact on coverage, with a decline of 5.8% in 2020 and 13.0% in 2021. In this region, vaccination coverage in the pandemic years was over 80% (87.2% in 2020 and 80.5% in 2021), close to what would be expected (92.6%) and much higher than the coverage observed in the north (67.9% in 2020 and 62.6% in 2021) and northeast (73.5% and 66.8%, respectively) (Figure 2).

**Figure 3:**
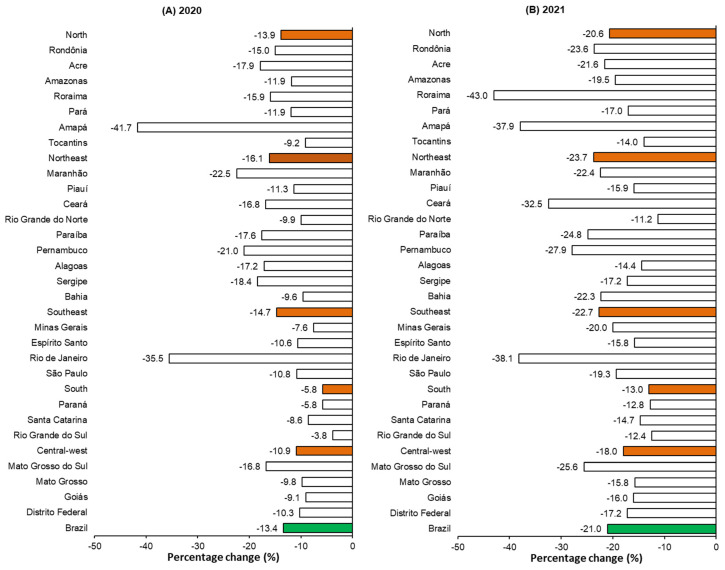
Impact of the COVID-19 pandemic on human rotavirus vaccination coverage during the COVID-19 pandemic in Brazil, according to Brazilian regions and states. Brazil, 2015–2021. Figures (**A**,**B**) show the impact of the COVID-19 pandemic on human rotavirus vaccination coverage by region and state in the years 2020 and 2021.

All Brazilian states showed a reduction in vaccination coverage. In 23 of them, the observed impact was greater in the year 2021 when compared to 2020. In the first year of the COVID-19 pandemic, the most significant reductions were observed in the states of Amapá (−41.7%) and Rio de Janeiro (−35.5%). In Amapá, for example, the coverage expected for 2020 and 2021 was 80.7%; however, the observed coverage was 47.1% in 2020 and 50.1% in 2021. In 2021, the impact was even more significant. In Roraima, also in the northern region, the observed coverage was 43% lower than expected for 2021 (expected = 88.9%; observed = 50.7%). The states of Rio de Janeiro (−38.1%), Amapá (−37.9%), and Ceará (−32.5%) also stand out; the reduction in each exceeded 30% (Figure 3).

**Figure 4:**
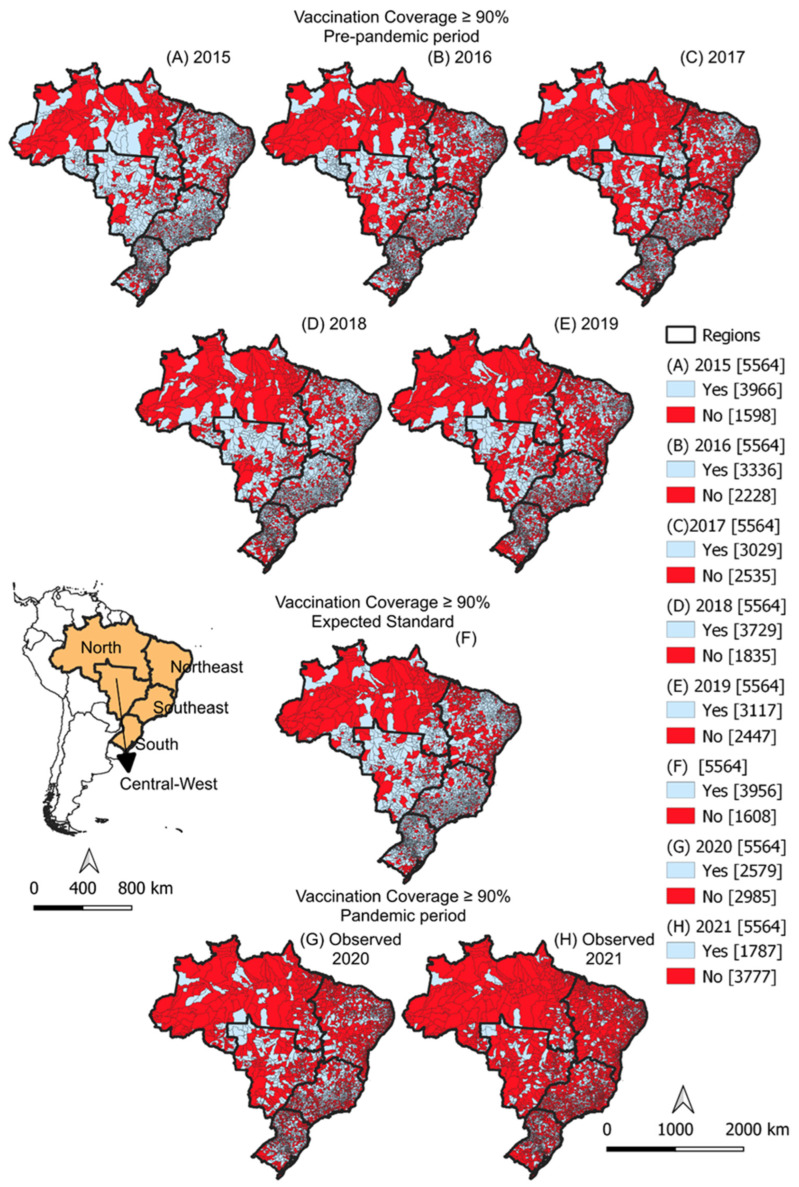
Achievement of human rotavirus vaccine coverage target (≥90%) before and during the COVID-19 pandemic in Brazil, according to Brazilian municipality. Brazil, 2015–2021. Figures (**A**–**E**) represent the situation of Brazilian municipalities regarding vaccination coverage (whether or not they reached the goal of coverage ≥ 90%). Figure (**F**) represents the minimum expected situation for 2020 and 2021. Figures (**G**,**H**) represent the situation observed in pandemic years.

At the municipal level, in the years prior to the COVID-19 pandemic, the proportion of Brazilian municipalities that reached the target vaccine coverage fluctuated: 71.3% in 2015, 60.0% in 2016, 54.4% in 2017, 67.0% in 2018, and 56.0% in 2019 (Figure 4A–E). Considering this previous coverage, it was expected that at least 71.1% (*n* = 3956) of municipalities would achieve more than 90% coverage in the years 2020 and 2021 (Figure 4F). However, it was observed that only 46.4% (*n* = 2579) reached the target in 2020 and 32.1% (*n* = 1787) did so in 2021 (Figure 4G–H).

**Figure 5:**
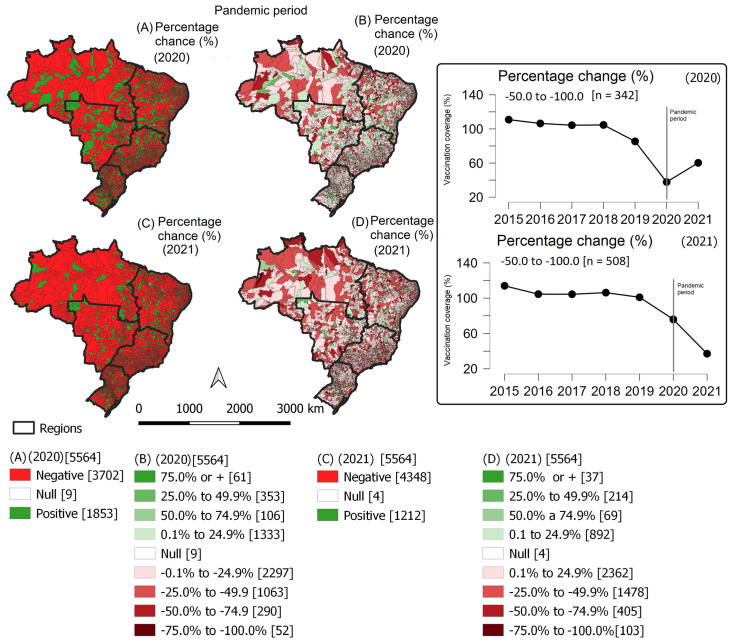
Impact of the COVID-19 pandemic on human rotavirus vaccination coverage during the first two years of the COVID-19 pandemic in Brazil, by municipality. Brazil, 2020–2021. Figures (**A**,**C**) show the classification of the percentage change in vaccination coverage in the years 2020 and 2021 (negative, null, or positive). Figures (**B**,**D**) show the classification of the percentage change in vaccination coverage.

When analyzing the type of impact (positive or negative) on vaccination coverage, 65.5% of municipalities (*n* = 3702) had reduced vaccination coverage in 2020, and 78.1% (*n* = 4348) did in 2021. Additionally, the proportion of municipalities with more than a 50% reduction almost doubled in 2021 when compared to the first pandemic year (342 in 2020 and 508 in 2021). In the 342 municipalities with the greatest reduction in 2020, vaccination coverage was 37.7%. In 2021, coverage increased again to 60.2%, much lower than the average of the years before the pandemic (102.3%) (Figure 5).

In the 508 counties with more than a 50% reduction in 2021, the pre-pandemic vaccination coverage was 106.0%. In 2020, it reduced to 75.9%, and in 2021, it was only 37.1%. It was expected that at least 76.6% (*n* = 389) of these municipalities would have reached the goal in 2021, but only two of them achieved more than 90% coverage (Ereré in the state of Ceará and Condor in the state of Rio Grande do Sul). Historically, these two municipalities had coverage higher than 100%, i.e., they vaccinated more children than estimated (Figure 5).

## 4. Discussion

The COVID-19 pandemic, associated with restrictive sanitary measures, negatively impacted rotavirus vaccination in Brazil. The reduction in vaccination coverage was more pronounced in the second year of the pandemic, although in this period, the mobility restrictions were already less rigid. Of the five Brazilian macro-regions, the northeast had the largest reduction, and the south had the smallest. Apart from the states of Amapá, Maranhão, Alagoas, and Bahia, the other 23 states had a greater impact in the year 2021 when compared to the year 2020. At the municipal level, fewer than half of the municipalities achieved vaccination coverage above 90% in either pandemic year.

In Brazil, the reduction in HRV vaccination coverage represented a significant drop from 2020 to 2021, decreasing from 77.3% to 70.4%. Other South American countries also evidenced a reduction in rotavirus vaccination in the year 2020 compared to pre-pandemic years: Argentina (77% in 2019 to 72% in 2020), Peru (90% in 2019 to 76% in 2020), Bolivia (78% in 2019 to 74% in 2020), and Colombia (92% in 2019 to 87% in 2020). In 2021, Argentina (78%) and Peru (82%) managed to recover some of their vaccination coverage, Colombia (86%) managed to maintain similar levels to the previous year, and Bolivia (71%) showed a reduction in coverage [27,28,29].

On the other hand, developed countries such as the United States and Canada have reported that overall vaccination coverage, after the initial decline in the first few months of the pandemic, returned to pre-pandemic levels by May 2020. In the United States, a reduction in the number of vaccine doses administered was detected in the second week of March 2020, but returned to levels comparable to 2019 by the third week of May [30,31]; thus, the reduction did not impact annual rotavirus vaccination coverage from 2020 (75%) to 2021 (76%) [28,29]. Canada also showed a similar pattern, with a drop in March and April and a recovery in May and June 2020 [13,32]. There was no impact on the annual rotavirus vaccination coverage, which remained at 84% in the two pandemic years [28,29]. In both countries, similar strategies were adopted in response to the vaccine decline, such as encouraging healthcare providers to request and provide routine vaccines, using reminders and recall tools, and highlighting the importance of vaccination at public press conferences.

It is important to highlight that in Brazil, since 2016, a decrease in vaccination coverage rates has been observed, with levels below the recommended vaccination goals for the vaccines included in the National Calendar [33]. This decreasing trend, which has been observed in recent years, is multifactorial and can be attributed to the confluence of several sociocultural, political, and personal factors, the main one being vaccine hesitancy. Political conflicts; partial unavailability of some products; operational problems for the proper execution of vaccination, including adequate data recording; and the difficulty of transporting vaccines to remote areas are some of the challenges to be overcome [33,34]. Reduced vaccination coverage rates signal a problem for collective immunity and a risk of resurgence of previously controlled or eradicated diseases [35].

In 2019, the WHO considered “vaccine hesitancy” as one of the top ten global health threats [36]. Vaccine hesitancy was defined as delaying the execution of the vaccination schedule or refusing to receive the recommended vaccines, despite their availability in health services [19]. This phenomenon originates from doubts about the real need for vaccines, fear of possible adverse events, concerns about safety and efficacy, and previous negative experiences with vaccination. It is propagated through the dissemination of fake news, especially on social networks, in the form of emotionally appealing phrases with no scientific evidence which generate panic and confusion [18]. Currently, one out of every five instances of fake news circulating in Brazil is regarding vaccines [37].

Although the Unified Health System (SUS, acronym in Portuguese) guarantees universal and equal access to immunization throughout the country, geographic heterogeneity and inequality among the regions of Brazil mean that vaccination coverage among regions and states is also unequal. The northern and northeastern regions were those with the lowest rotavirus vaccination coverage, even with an increasing trend in the prevalence of vaccine availability in these regions [38]. Both regions, due to high social vulnerability, were also the most affected by the COVID-19 pandemic, registering the highest lethality rates associated with the disease [39,40]. The states most impacted by the pandemic in terms of rotavirus vaccination coverage in 2020 (Amapá, Rio de Janeiro, and Maranhão) and 2021 (Roraima, Rio de Janeiro, and Amapá) belong to the northern and northeastern regions, except for Rio de Janeiro, which belongs to the southeastern region.

The population of the northeast is exposed to an environment with greater vulnerability (poverty, less access to public health services, and less primary care coverage) when compared to the south, for example. In poorer regions, the pandemic caused more severe damage than that observed in richer regions. The context of social vulnerability explains the greater negative impact in the northeast [40]. This finding indicates that this region should be the target of more intense intervention in the coming years in order to mitigate the damage caused by the COVID-19 pandemic.

Different investigations have shown that the burden of the COVID-19 pandemic was greater in more vulnerable areas. Souza et al. [40] showed that the COVID-19 mortality rate in municipalities with very high social vulnerability was higher than that observed in those municipalities with very low social vulnerability. Additionally, social determinants that express vulnerability were associated with higher mortality, such as low per capita income, low educational levels, and poor access to public services.

The Ministry of Health has established adequate vaccine coverage for HRV as greater than or equal to 90%. However, the homogeneity of vaccine coverage among municipalities is also an important performance indicator of the National Immunization Program, and was established in at least 70% of municipalities with adequate vaccine coverage [24]. Only in 2015 did Brazil manage to achieve this goal, with 71.3% of municipalities in the country having coverage above 90%. Since 2016, a gradual drop in homogeneity has been observed, except for the year 2018, which recorded an increase. In the pandemic years, there was a sharp drop in 2020 (46.4%) that intensified in 2021 (32.1%).

Rotavirus vaccination has had a significant positive impact in the areas with the worst socioeconomic indicators in Brazil, accounting for a significant reduction in hospitalization for diarrhea and its mortality rates since 2006 [41]. However, the areas with the greatest vulnerability were also those most affected by the COVID-19 pandemic [40] and those who experienced a significant decline in HRV vaccination coverage in the pandemic years. However, the Brazilian government did not implement interventions to maintain or increase vaccination coverage rates during the pandemic. On the contrary, the government’s approach to dealing with the pandemic at the federal level was considered tragic [42,43].

Within the Unified Health System, Primary Health Care (PHC) is the main entity responsible for implementing the PNI, with the role of storing, applying, and monitoring the coverage of immunobiologicals [9]. The PHC is responsible for applying the vaccine to the Brazilian population. There are more than 38,000 vaccine rooms distributed throughout the national territory. In this sense, the PHC has a fundamental role in facing diseases preventable by immunizers, such as rotaviruses and COVID-19 [9,22,44,45,46]. Additionally, the PHC is responsible for approximately 95% of the vaccines administered nationwide [9].

Primary care also has a central role in emergency preparedness and response, such as the COVID-19 pandemic, given its high degree of capillarization in the national territory and the fact that it reached expressive portions of the population exposed to excessive risks due to their living conditions [44,45,46]. The importance of PHC, however, has not had due recognition when one observes the problems in the structures of basic health units (BHUs), the lack of materials and inputs, and the lack of vaccine supply (in 2018, about a quarter of BHUs did not have all vaccines) [38].

For this reason, in a country of continental dimensions such as Brazil, which is characterized by a polarized epidemiological transition process both geographically and nosologically, the strengthening of PHC emerges as the most important action capable of guaranteeing the right to health, as prescribed in the Brazilian Constitution of 1988. Thus, it is not possible to reduce inequalities without strengthening PHC.

Even considering the methodological care, this study has limitations that should be considered when interpreting the results. First, this is an ecological evaluation of secondary data obtained from the Immunization Program Evaluation System, so there is the possibility of bias regarding the quantity and quality of information. The immunization numbers are manually entered into the system by the municipal health secretariats and are subject to errors and delays in their completion, especially in those localities with deficient surveillance systems.

It is important to note that the clinical impact of reduced vaccination coverage on hospitalizations or mortality from rotaviruses, for example, is not known. For this reason, this is an issue that requires further investigation since it was not the subject of this study. In addition, it should be noted that other factors directly or indirectly associated with the COVID-19 pandemic must also be considered. As stated earlier, vaccine hesitancy was already a worldwide phenomenon, but it may have been accentuated during the COVID-19 pandemic.

## 5. Conclusions

In Brazil, a severe impact of the COVID-19 pandemic on rotavirus vaccination coverage was observed. The decline in vaccination coverage had already been observed, but it showed a significant drop in 2020 which deepened in 2021.

Maintaining vaccination activities, ensuring the necessary supplies and materials, and making adequate amounts of vaccines available must be a priority for the government and health managers. Monitoring of vaccination coverage in the coming years must be carried out continuously in order to avoid a possible resurgence of rotavirus-induced diarrhea, a vaccine-preventable disease.

## Data Availability

Not applicable.
